# Distribution of picophytoplankton communities from brackish to hypersaline waters in a South Australian coastal lagoon

**DOI:** 10.1186/1746-1448-6-2

**Published:** 2010-02-24

**Authors:** Mathilde Schapira, Marie-Jeanne Buscot, Thomas Pollet, Sophie C Leterme, Laurent Seuront

**Affiliations:** 1 School of Biological Sciences, Flinders University, GPO Box 2100, Adelaide SA 5001, Australia; 2 Southern Ocean Group, Department of Zoology & Entomology, Rhodes University, PO box 94, Grahamstown 6140, South Africa; 3 UMR CARRTEL, Centre Alpin de Recherche sur les Réseaux Trophiques des Ecosystèmes Limniques, Station d'Hydrobiologie Lacustre, Université de Savoie, 75 avenue de Corzent, BP 511, 74203 Thonon les Bains Cedex, France; 4 South Australian Research and Development Institute, Aquatic Sciences, West Beach SA 5022, Australia; 5 Center for Polymer Studies, Department of Physics, Boston University, 590 Commonwealth Avenue, Boston, MA 02215 USA

## Abstract

**Background:**

Picophytoplankton (i.e. cyanobacteria and pico-eukaryotes) are abundant and ecologically critical components of the autotrophic communities in the pelagic realm. These micro-organisms colonized a variety of extreme environments including high salinity waters. However, the distribution of these organisms along strong salinity gradient has barely been investigated. The abundance and community structure of cyanobacteria and pico-eukaryotes were investigated along a natural continuous salinity gradient (1.8% to 15.5%) using flow cytometry.

**Results:**

Highest picophytoplankton abundances were recorded under salinity conditions ranging between 8.0% and 11.0% (1.3 × 10^6 ^to 1.4 × 10^6 ^cells ml^-1^). Two populations of picocyanobacteria (likely *Synechococcus *and *Prochlorococcus*) and 5 distinct populations of pico-eukaryotes were identified along the salinity gradient. The picophytoplankton cytometric-richness decreased with salinity and the most cytometrically diversified community (4 to 7 populations) was observed in the brackish-marine part of the lagoon (i.e. salinity below 3.5%). One population of pico-eukaryote dominated the community throughout the salinity gradient and was responsible for the bloom observed between 8.0% and 11.0%. Finally only this halotolerant population and *Prochlorococcus*-like picocyanobacteria were identified in hypersaline waters (i.e. above 14.0%). Salinity was identified as the main factor structuring the distribution of picophytoplankton along the lagoon. However, nutritive conditions, viral lysis and microzooplankton grazing are also suggested as potentially important players in controlling the abundance and diversity of picophytoplankton along the lagoon.

**Conclusions:**

The complex patterns described here represent the first observation of picophytoplankton dynamics along a continuous gradient where salinity increases from 1.8% to 15.5%. This result provides new insight into the distribution of pico-autotrophic organisms along strong salinity gradients and allows for a better understanding of the overall pelagic functioning in saline systems which is critical for the management of these precious and climatically-stress ecosystems.

## Background

The ubiquitous distribution of picophytoplankton and their importance in terms of biomass and production, make them a critical component of food web and carbon cycling in marine systems [1-3]. In particular the partitioning between picophytoplankton and larger cells reflects the source and cycling of nutrients [4] and influences the pathway of matter transfer to higher trophic levels [5].

Flow cytometry has been extensively used to investigate the distribution of phototrophic picoplankton and three groups may be identified in unstained samples: *Prochlorococcus *sp., *Synechococcus *sp. and pico-eukaryotic phototrophs [6]. Environmental factors controlling the distribution and composition of these distinct communities have been extensively reviewed, such as light requirement [5,7], water temperature [8,9], nutrient levels [1,10], grazing [11,12] and viral lysis [13,14]. However, most of these investigations concerned pelagic ecosystems and the picophytoplankton communities in coastal waters have still received little attention.

Coastal habitats are characterized by strong environmental gradients which are likely to be important areas of highly dynamic compositional and functional changes [15]. In particular, important ecological changes such as decreasing biodiversity and increasing dominance of prokaryotes are assumed to occur along salinity gradients [16]. However, little is still known about the effect of salinity on the distribution and community composition of picophytoplankton [17].

Several studies have been performed within estuaries or bays investigating planktonic cyanobacteria and/or eukaryotic picophytoplankton communities [e.g. [14,18,19]]. However, in these studies, salinity never exceed 3.5% and the dynamics of phototrophic communities under high salinity conditions (i.e. above 3.5%) has been mainly investigated in crystallizer ponds from solar salterns [20-22] or in hypersaline lakes [17,23,24].

In this context, the present study investigates the distribution of picophytoplankton (i.e. cyanobacteria and eukaryotes) communities along a strong and continuous salinity gradient. With salinity gradually increasing from brackish (1.8%) to hypersaline (15.5%), the Coorong, a shallow South Australian lagoon, represents a unique model system to investigate the role of salinity in shaping the niche development in picophytoplankton communities. This shallow coastal lagoon is one of Australia's most significant wetlands especially as a waterbird habitat and has been designated a wetland of international importance under the Ramsar Convention in 1985. Over the past decades, this system has been impacted by the building of barrages that favored the flow of seawater into the wetlands over the usual freshwater flow from the river Murray. In addition, climate variability (lower freshwater inputs and higher evaporation processes) also led to increase the salinity of the lagoon. A better knowledge of the picoplankton communities inhabiting the different part of the lagoon is thus essential both locally, for the management of this fragile ecosystem, and globally as a unique model system to deeply investigate the potential consequences of environmental changes and perturbations on community shifts.

More specifically, given the lack of information related to the dynamics of picophytoplankton communities along continuous natural hypersaline gradients, our objectives were to (i) investigate the changes in abundance and diversity of flow cytometrically-defined populations of planktonic cyanobacteria and pico-eukaryotes along the salinity gradient and (ii) identify the main factors driving their distributions.

## Methods

### Study site

**Figure 1:**
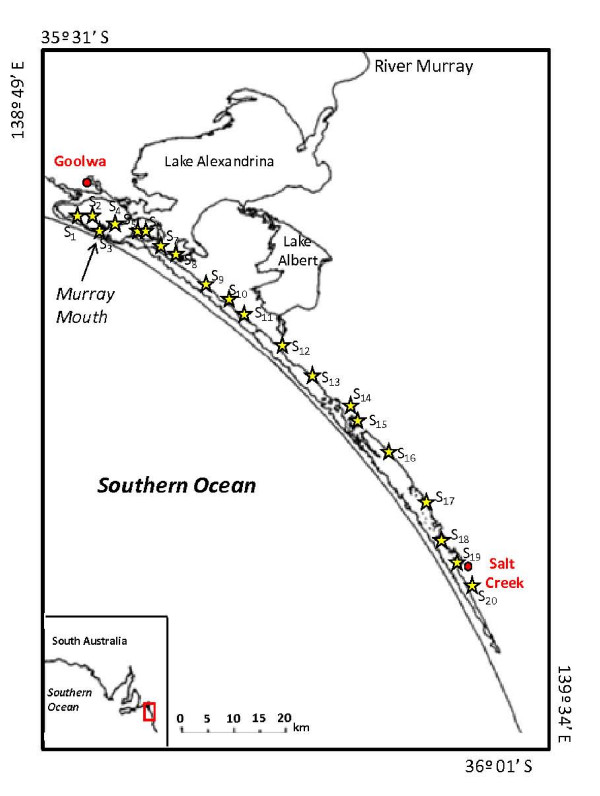
**Study area, the Coorong (South Australia)**. This shallow coastal lagoon, located approximately 90 km south-east of Adelaide in South Australia, is bordered by adjacent fresh water lake (Lake Alexandrina and Lake Albert) and separated from the open ocean by a network of sand dunes. This coastal lagoon forms the Murray Mouth with the lower lakes, which is the terminal lake system of the River Murray. More than 140 km in length, the Coorong is characterised by a strong salinity gradient with salinity values ranging from ca. 2.0% close to the Murray Mouth to more than 15.0% near Salt Creek. Location of the 20 sampling sites (black star; S_1 _from S_20_).

The Coorong is a shallow lagoon in South Australia, parallel to the coast and separated from the open ocean by a network of sand dunes (Fig. 1). This coastal lagoon forms the Murray Mouth with the lower lakes (Lake Alexandrina and Lake Albert), which is the terminal lake system of the River Murray [25]. More than 140 km in length, the Coorong is characterised by a strong salinity gradient with salinity values ranging from ca. 2.0% close to the Murray Mouth to more than 15.0% near Salt Creek (Fig. 1). As the saline waters of the Coorong receive inputs from the ocean through the Murray Mouth and from underground, the concentration of salts along the lagoon is similar to seawater. The saline waters of the lagoon are separated from the lower lakes by a series of barrages. The freshwater inputs through the barrages lead to lower salinities in the North-West part of the Coorong, whereas the excess in evaporation over precipitation increases salinity along its North-South axis, especially during the summer period characterised by (i) low water levels (ranging from 0.5 m near the Murray Mouth to 0.9 m in the southern part of the lagoon) and (ii) weak tidal impact [25].

### Sampling

Samples were collected at 20 locations along the lagoon, from the brackish waters near Goolwa (salinity = 1.8%) to the hypersaline waters near Salt Creek (salinity = 15.5%; Fig. 1) on February 3-4, 2007. At each sampling site, referred to as S_i _with *i *= 1 to 20 (Fig. 1), measurements and water sample collection were performed in 50 cm of water (a depth representative of most parts of the lagoon) from (i) the sub-surface waters and (ii) at the water-sediment interface (WSI). Temperature (°C), conductivity (mS cm^-1^) and dissolved oxygen concentrations (DO; mg l^-1^) were recorded using a YSI 85 (Fondriest) multiparameter probe. Salinity (%) was calculated from temperature and conductivity following Fofonoff and Millard [26]. 1 liter water samples were collected at each depth using acid-washed 1-liter borosilicate bottles with special care to avoid sediment resuspension.

Dissolved inorganic nutrient concentrations were determined from 12 ml filtered (Whatman GF/C) water samples. Analyses were performed in the field using a portable LF 2400 photometer (Aquaspex^®^) according to standard colorimetric methods for  (Indophenol blue),  (Naphtylethylene diamine),  (Naphtylethylene diamine after zinc reduction) and  (Ascorbic acid reduction). Ammonium, nitrite, nitrate and phosphate measures ranged from 0.6 to 110 μM, 0.2 to 160 μM, 1.6 to 160 μM and 1.1 to 50 μM, respectively. Samples (50 to 100 ml) for suspended particulate material concentration (SPM; mg l^-1^) were filtered through pre-combusted (400°C; 4 hours) and pre-weighted glass-fibre filters (Whatman GF/C; pore size = 1.2 μm), and immediately deep frozen in liquid nitrogen until analysis. In the laboratory, filters were rinsed with MilliQ water, dried at 60°C for 24 h, and reweighed to determine the mass of suspended solid retained on the filter [27].

Two distinct set of samples (3 × 1 ml) were collected for the identification and enumeration of virus-like particles and picophytoplankton populations by flow cytometry.

### Virus-like particles

Virus-like particles (VLP) were identified through flow cytometry to assess the presence of phytoplankton phages that could potentially infect phytoplankton. These phytoplankton phages can be discriminated from the other groups of virus by their higher side scatter (related to their size) and/or green fluorescence (related to their DNA content) as previously shown in recent works [28,29]. Samples were collected in triplicate (1 ml) at each sampling station, fixed with 0.5% (final concentration) glutaraldehyde in the dark at 4°C for 15 min, quick frozen in liquid nitrogen and then stored at -80°C until analysis. After being quick thawed, samples were diluted (1:10) in 0.2 μm filtered TE Buffer stained with SYBR Green I solution (1:5000 dilution) and incubated at 80°C in the dark for 10 min [30].

### Phytoplankton biomass

Phytoplankton biomass was estimated through chlorophyll *a *(Chl *a*) concentrations. Samples (50 to 100 ml) were filtered through glass-fibre filters (Whatman GF/C) and immediately deep frozen in liquid nitrogen until analysis. Chlorophyllous pigments were then extracted in 5 ml of methanol in the dark at 4°C during 24 h [31]. Concentration of Chl *a *(μg l^-1^) was determined following Strickland and Parson [32] using a Turner 450 fluorometer previously calibrated with a pure Chlorophyll *a *solution (*Anacystis nidulans *extract, Sigma Chemicals, St Louis).

### Picophytoplankton abundances

Photosynthetic picophytoplankton populations were identified and enumerated by flow cytometry (FCM) using a FACScanto flow cytometer (Becton-Dickinson) equipped with an air-cool argon laser (15 mW, 488 nm) with phosphate buffer saline (PBS) solution employed as a sheath fluid. Water samples (1 ml) were fixed in the field with 2% (final concentration) of paraformaldehyde, immediately deep frozen in liquid nitrogen and then stored at -80°C.

After being quick thawed, picoplankton cells were discriminated and enumerated by FCM according to their specific auto-fluorescence and light scatter properties [33,34]. Forward-angle light scatters (FSC), right-angle light scatter (SSC), red and orange fluorescence, were recorded for each sample. As the values of FSC are those most affected by density differences between the sheath fluid and the samples [21], the values of FSC were not used to enumerate cells. Fluorescent beads 1 μm in diameter (Molecular Probes, Eugene, Oregon) were added to all samples as an internal standard. Working beads concentrations were estimated after each FCM session under epifluorescent microscopy to ensure reliability of the beads concentration and all FCM parameters were normalized to bead concentration and fluorescence. Finally, picophytoplankton populations were identified and enumerated using WinMDI 2.9 (^©^Joseph Trotter) flow cytometry analysis software. *Synechococcus *sp., *Prochlorococcus *sp. and autotrophic pico-eukaryotic cells were discriminated in plots of SSC versus orange fluorescence (from phycoerythrin) and red fluorescence (from chlorophyll), according to standards protocols [33,34]. *Synechococcus *and *Prochlorococcus *cells can be discriminated by their flow cytometry scatter signal (SSC) related to their size, and their fluorescence emission when excited by a blue light. The phycobilins contained in *Synechococcus *emit a strong orange fluorescence, whereas *Prochlorococcus *harvest light mainly through chlorophyll *a *and *b*, and therefore emit only red fluorescence when excited by blue light [35]. In addition, *Synechococcus *cells are larger than *Prochlorococcus *cells (ca. 1-0.6 μm in diameter respectively) [4]. Pico-eukaryotes were identified by their larger size (SSC) and higher red fluorescence. Because, in the absence of genetic fingerprinting the identification of *Synechococcus *and *Prochlorococcus *cannot be warranted *sensu stricto*, the populations exhibiting the flow cytometric signatures of *Synechococcus *and *Prochlorococcus *as reported in the literature were referred to as *Synechococcus*-like and *Prochlorococcus*-like populations.

### Data analysis

Comparisons between the two sampling depths were conducted using the Wilcoxon-Mann-Whitney *U*-test (*U*-test hereafter). The BIOENV [36] and BVSTEP [37] procedures (PRIMER version 6.0) were used to investigate relationships between environmental variables and picophytoplankton community's composition along the salinity gradient. Both analysis compare rank correlation between the matrices of environmental variables (based on normalised Euclidian distance) and the biotic similarity matrix of picophytoplankton variables (based on the Bray-Curtis similarity) using different permutations of the environmental variables. BIOENV compares different combinations of a specified number of variables, whereas BVSTEP uses a stepwise procedure to identify the best subset of variables. Spearman rank correlations between the biotic and abiotic similarity matrices were used to identify the best suites of environmental variables that best explained the distribution of picophytoplankton communities along the salinity gradient and the significance of the correlation was determined using a permutation procedure [38]. Environmental variables considered in the BIOENV/BVSTEP analysis were salinity, [DO], [SPM], [], [ + ], [] and VLP3 abundances. As temperature variability between stations was mainly related to the time of the day when the sampling occurred, this parameter was not considered in the analyses. Similarities between stations for picophytoplankton communities along the salinity gradient were inferred through a cluster analysis (i.e. hierarchical agglomeration using complete linkage cluster analysis performed on Euclidian distances) performed on the log (abundance + 1) data matrix [39]. This analysis was performed with STATISTICA version 8 software.

## Results

No significant differences were found between sub-surface and water-sediment interface (WSI) for any abiotic or biotic parameters (*U*-test, 0.10 < P < 0.02). This indicates that the water column was well mixed along the lagoon, in accordance with previous results [25]. Sub-surface and WSI data were then pooled for further analysis.

### Environmental parameters

Water temperature ranged between 25.2°C and 27.7°C. Salinity increased from 1.77% in S_1 _to 15.48% in S_20 _(sampling site, referred to as S_i _with *i *= 1 to 20, see Fig. 1, Table 1). At stations S_1 _and S_2_, salinity levels remained below 2.50%. Salinity then slowly increased from 2.75% to 5.03% between stations S_3 _and S_11_. In contrast, salinity sharply increased from station S_12 _to reach 15.0% at station S_17 _(Table 1).

**Table 1:** Hydro-chemical parameters along the salinity gradient.

Station	Salinity (%)		**+ **	
		(μM)	(μM)	(μM)
S_1_	1.77	91.7	*< DL*	40.0
S_2_	2.44	56.0	*< DL*	35.3
S_3_	2.74	20.3	*< DL*	32.6
S_4_	3.24	22.5	*< DL*	21.6
S_5_	3.28	28.3	*< DL*	48.4
S_6_	3.32	60.0	*< DL*	20.0
S_7_	3.35	14.2	1.8	4.7
S_8_	3.54	26.1	*< DL*	*< DL*
S_9_	4.27	10.3	2.0	*< DL*
S_10_	4.69	6.7	*< DL*	3.2
S_11_	5.03	13.3	*< DL*	*< DL*
S_12_	6.75	16.7	*< DL*	2.1
S_13_	8.30	48.9	2.0	9.0
S_14_	10.00	72.5	*< DL*	17.9
S_15_	10.72	78.2	*< DL*	11.1
S_16_	13.35	*> R*	*< DL*	16.3
S_17_	15.01	*> R*	1.6	*< DL*
S_18_	14.92	*> R*	*< DL*	*< DL*
S_19_	14.54	*> R*	*< DL*	3.7
S_20_	15.48	*> R*	1.6	3.7

Salinity (%), ammonium [], nitrite + nitrate [ + ] and phosphate [] concentrations (μM) observed on the 20 sampling stations (S_1 _to S_20_). DL: detection limit. R: maximum range. Ammonium, nitrite, nitrate and phosphate measures ranged from 0.6 to 110 μM, 0.2 to 160 μM, 1.6 to 160 μM and 1.1 to 50 μM, respectively.

Ammonium ([]) was by far the most abundant form of nitrogen and represented more than 80% of the total inorganic nitrogen pool throughout the salinity gradient (Table 1). [] concentrations increased with salinity and highest concentrations (i.e. > 110 μM) were observed from 13.35% (S16; Table 1). Phosphate concentrations []) were relatively high below 3.32% (i.e. from S_1 _to S_6_) with values ranging between 20 to 40 μM, and decrease thereafter to reach very low levels (i.e. <4 μM) between 3.35% and 6.75% (i.e. from S_7 _to S_12_). Relatively high concentrations (i.e. 9 to 16 μM) were observed again from 8.30% to 13.35% (i.e. S_13 _to S_16_) and decreased thereafter to reach very low level (i.e. < 4 μM) in the hypersaline waters of the lagoon (i.e. salinity > 14.0%; Table 1).

**Figure 2:**
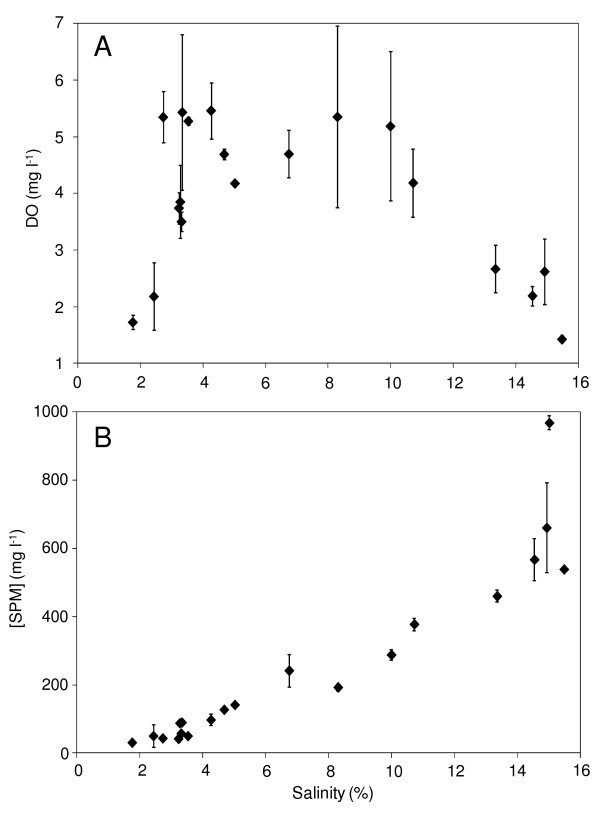
**Dissolved oxygen and suspended particular matter along the salinity gradient**. (A) Dissolved oxygen (DO; μmg l l^-1^) and (B) suspended particular matter (SPM; mg l^-1^) concentrations along the salinity gradient (salinity; %). The error bars are the standard deviations.

Dissolved oxygen (DO) concentrations ranged between 1.4 and 5.5 mg l^-1 ^along the salinity gradient (Fig. 2A). DO concentrations remained below 3.0 mg l^-1 ^in the brackish (i.e. salinity < 2.5%) and hypersaline waters of the lagoon (i.e. salinity > 13%). Highest DO values, ranging between 4.2 and 5.5 mg l^-1^, were observed between 2.74% and 10.72% (Fig. 2A). Concentrations of suspended particulate matter (SPM) increased exponentially along the salinity gradient, with values increasing from 30 mg l^-1 ^at 1.77% to 967 mg l^-1 ^at 15.01% (Fig. 2B).

### Virus-like particles

**Figure 3:**
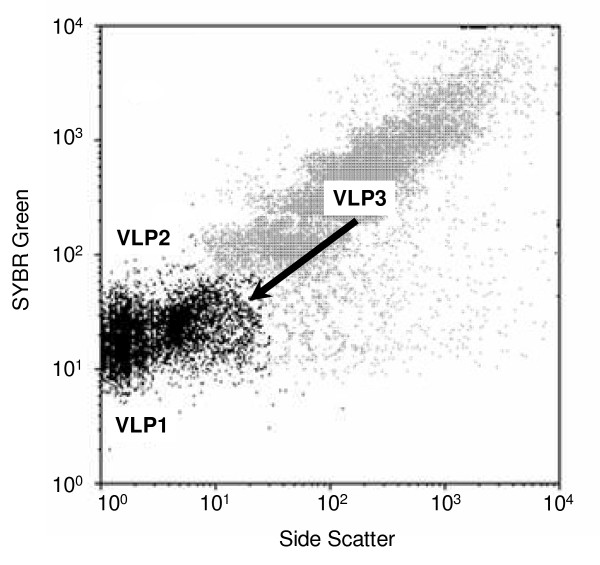
**Cytometric differentiation of virus-like particles**. Scatter plot of side scatter (SSC) versus green fluorescence (SYBR Green) showing 3 viral sub-populations: VLP1, VLP2 and VLP3. Sub-populations of virus-like particles (VLP) were discriminated based on their differences in SYBR Green fluorescence and SSC, as non-overlapping classes of size and green fluorescence according to Brussaard [30]. VLP1 and VLP2 correspond to populations observed previously in sea water samples and described as bacteriophages [33]. The sub-population VLP3 exhibited the same SYBR Green fluorescence level than VLP2 but was characterized by higher SSC. Recent work have indicated that viral population presenting a cytometric signature similar to the one observed here for VLP3, were likely to be phytoplankton viruses [28,29].

Three virus-like particles (VLP) populations were identified along the salinity gradient (Fig. 3). Details of the pattern of VLP populations along the salinity gradient are fully described in Schapira *et al*. [40]. Briefly, the two first populations (VLP1 and VLP2) exhibit the same cytometric signature (SSC and green fluorescence) of viral population observed previously in seawater and identified as bacteriophages [33]. In contrast, the third population (VLP3) exhibits the same SYBR Green fluorescence (related to DNA content) level as VLP2 but a higher side scatter (i.e. size; Fig. 3), hence could represent a group of phytoplankton viruses [28,29]. VLP3 was only observed from 5.03% (S_11_) to 10.00% (S_14_) with abundances ranging between 1.9 × 10^6 ^and 4.3 × 10^6 ^VLP ml^-1^.

### Phytoplankton biomass

**Figure 4:**
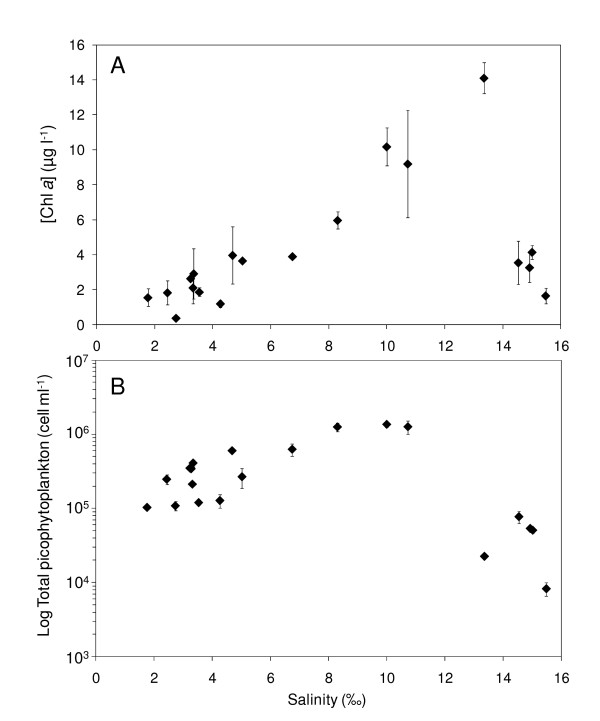
**Phototrophic organisms along the salinity gradient**. (A) Phytoplankton biomass [Chl *a*] (μg l^-1^) and (B) abundances of total picophytoplankton cells (cell ml^-1^; in log scale) along the salinity gradient (salinity; %). The error bars are the standard deviation.

Chlorophyll *a *(Chl *a*) concentration increased from 0.4 μg l^-1 ^to 14.1 μg l^-1 ^with salinity increasing from 1.77% to 13.36% (Fig. 4A). Chl *a *concentrations sharply decreased thereafter, for salinity greater than 14.0%, and remained below 4.5 μg l^-1 ^in the hypersaline part of the lagoon (Fig. 4A).

### Picoplankton abundance and community structure identified using FCM

**Figure 5:**
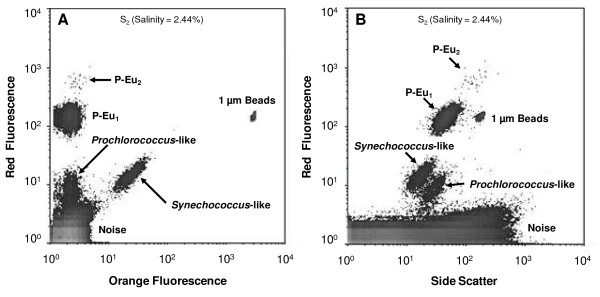
**Cytometric differentiation of picophytoplankton populations**. These different subpopulations were identified according to their differences in side scatter and both red and orange fluorescence; *Synechococcus *emit a strong orange fluorescence whereas *Prochlorococcus *emit only red fluorescence when excited by blue light [35]. In addition, *Synechococcus *cells are significantly larger than *Prochlorococcus *cells (ca. 1-0.6 μm in diameter respectively) [4]. Pico-eukaryotes were identified by their larger size (SSC) and higher red fluorescence. Scatter plot of orange versus red fluorescence (A) and side scatter versus red fluorescence (B) observed on Station S_2 _(i;e. Salinity = 2.44%), showing: (i) 2 populations of pico-cyanobacteria, one exhibiting fluorescence and side-scatter characteristics of *Prochlorococcus *sp. (referred as *Prochlorococcus*-like) and the second exhibiting fluorescence and side-scatter characteristics of *Synechococcus *(referred as *Synechococcus*-like) and (ii) 2 populations of pico-eukaryotes (*P-Eu*_1 _and *P-Eu*_2_).

Picophytoplankton abundances were highly variable along the salinity gradient with values ranging between 8.3 × 10^3 ^cells ml^-1 ^and 1.4 × 10^6 ^cells ml^-1 ^(Fig. 4B). Concentrations were relatively low (i.e. ≤ 6.3 × 10^5 ^cells ml^-1^) where salinity remained below 7.0% (Fig. 4B). High abundances were observed for salinity between 8.0% and 11.0% with values ranging from 1.3 × 10^6 ^cells ml^-1 ^to 1.4 × 10^6 ^cells ml^-1 ^(Fig. 4B). Above 13.0% picophytoplankton abundance was very low with values remaining below 8.0 × 10^4 ^cells ml^-1 ^(Fig. 4B). Samples were characterised by a highly complex community structure with multiple subpopulations of picophytoplankton throughout the salinity gradient. Flow cytometry analysis (FCM) revealed 2 distinct populations of pico-cyanobacteria (*P-Cya*) exhibiting fluorescence and side-scatter characteristics of *Prochlorococcus *sp. and *Synechococcus *sp. (referred hereafter as *Prochlorococcus*-like and *Synechococcus*-like), and 5 different populations of pico-eukaryotes (*P-Eu*), exhibiting consistently different side scatter (related to size) and red fluorescence. These different subpopulations were identified according to their differences in size and both red and orange fluorescence (Fig. 5). 1 μm beads were added in all occasion during flow cytometry run as a reference of size and the largest pico-eukaryote population was only slightly bigger than the beads (i.e. 1 μm).

**Figure 6:**
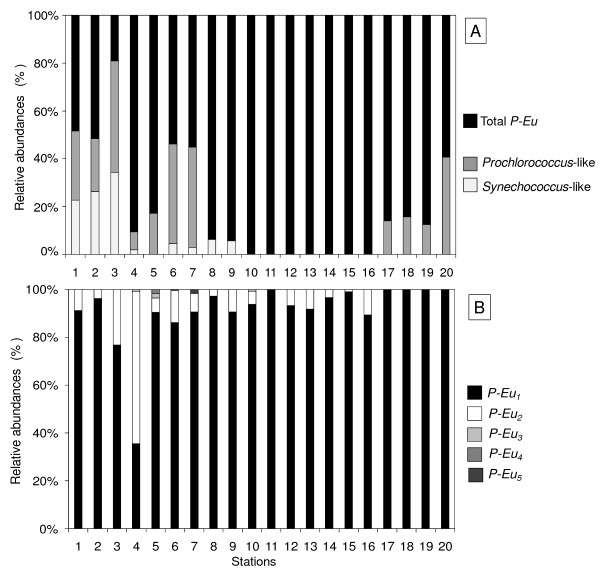
**Picophytoplankton communities along the salinity gradient**. Relative abundances (%) of cytometrically defined different picophytoplankton populations observed on the 20 sampling stations (S_1 _from S_20_). (A) Relative abundances of total pico- eukaryotes and cyanobacteria (i.e. *Prochlorococcus*-like. and *Synechococcus*-like). (B) Relative contribution of cytometrically defined different pico-eukaryotes populations (*P-Eu*_1_, *P-Eu*_2_, *P-Eu*_3_, *P-Eu*_4 _and *P-Eu*_5_) to the total pico-eukaryotes community.

**Figure 7:**
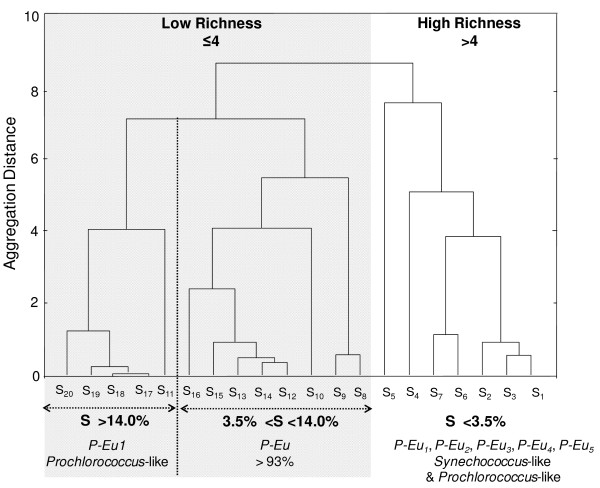
**Cluster analysis of picophytoplankton communities along the salinity gradient**. The analysis was performed on the log (abundance + 1) data matrix. The *x*-axis shows station from S_1 _to S_20_. Two main groups of stations were discriminated based on their population richness: high richness (white, >4 populations) for salinity <3.5% where the seven different sub-populations were identified (*P-Eu*_1_, *P-Eu*_2_, *P-Eu*_3_, *P-Eu*_4_, *P-Eu*_5_, *Synechococcus*-like and *Prochlorococcus*-like), and low richness for salinity >3.5% (grey, ≤ 4 populations). In this latter group, two sub-groups of stations where discriminated based on their population composition: a sub-group characterized by a great dominance of pico-eukaryotes (>93%) for salinity ranging between 3.5 and 14.0%, and a sub-group where only *Prochlorococcus*-like and *P-Eu*_1 _occurred for salinity greater than 14.0%.

Except in locations where salinity remained below 3.0%, *P-Eu *were by far the most abundant and contribute to more than 54% of the total abundances throughout the salinity gradient (Fig. 6A). Furthermore, the picophytoplankton community was only composed of *P-Eu *for salinities ranging from 4.5% to 14.0% (Fig. 6A). Below 3.0% (S_1 _to S_3_) *P-Cya *were much more abundant, contributing from 48% (S_2_) to 81% (S_3_) of the total community, with *Synechococcus-*like and *Prochlorococcus*-like populations respectively representing 23% to 34% and 22 to 47% of the total abundances (Fig. 6A). The *Synechococcus*-like population was observed from 1.7% to 4.5% (S_1 _to S_9_). In contrast, the *Prochlorococcus*-like population was observed for salinity ranging from 1.7% to 3.5% (S_1 _to S_7_) and for salinity greater than 14.0% (S_17 _to S_20_; Fig. 5, 6A). Five different sub-populations of *P-Eu *were discriminated (Fig. 6B). These populations consistently exhibited different side scatter and red fluorescence signatures (Fig. 5). *P-Eu*_1 _was by far the most abundant and contributed to more than 70% of the *P-Eu *abundances (Fig. 6B). *P-Eu*_2 _was the second most abundant population with relative contribution to the total *P-Eu *only occasionally exceeding 23% (Fig. 6B). Whilst *P-Eu*_1 _was identified throughout the salinity gradient *P-Eu*_2 _was not observed for salinity greater than 14.0%; *P-Eu*_1 _was also the only sub-population observed in these hypersaline waters of the lagoon (Fig. 6B). The relative abundances of populations *P-Eu*_3_, *P-Eu*_4 _and *P-Eu*_5 _remained below 2% and were locally observed mostly for salinity ranging from 3.2 and 3.5% (Fig. 6B). The cluster analysis performed on picophytoplankton abundance discriminated 2 main groups of stations based on their population richness (defined here as the cytometrically-defined richness, i.e. FCM richness): a high FCM richness group (i.e. richness ≥ 4) occurred for salinity lower than 3.5% (S_1 _to S_7_), and a low FCM richness group (i.e. richness < 4) included stations where salinity was greater than 3.5% (Fig. 7). In the later, two sub-groups of stations were identified: a sub-group where *P-Eu *(*P-Eu*_1_, *P-Eu*_2 _and *P-Eu*_3_) contributed to more than 93% to the total abundances occurring for salinity ranging between 3.5% and 14.0% (S_8 _to S_16_), and a sub-group characterized by a community composed exclusively by the *P-Eu *sub- population *P-Eu*_1 _and *Prochlorococcus*-like picocyanobacteria comprising stations where salinity was greater than 14.0% (S_17 _to S_20_; Fig. 7).

### Picophytoplankton and environmental variables

Multivariate correlations analysis, BIOENV showed that the environmental variable that best explained the picophytoplankton abundance pattern along the lagoon was salinity (ρ = 0.542; P < 0.01; Table 2). Salinity in combination with ammonium concentrations and abundance of viral population (VLP3) are the best subset of variables explaining the variability of picophytoplankton abundances observed along the salinity gradient (BVSTEP; Table 2). The Spearman's rank correlation coefficient for this analysis was 0.520 and was statistically significant (P < 0.01; Table 2).

**Table 2:** Results of the BIOENV and BVSTEP analyses.

	*n*	*ρ*	*P*	*k*	Environmental variables
BIOENV	9	0.542	0.01	1	S

BVSTEP	9	0.520	0.01	3	S - [] - VLP3

The table shows the combination of the best environmental variables that predict the patterns of picophytoplankton abundances along the salinity gradient. *n*: number of abiotic variables used in the analysis: *ρ *Spearman rank correlation coefficient; *P*: significance levels; *k*: number of corresponding significant environmental variables. Significant contributing environmental variables were ordered according to the degree of match. "S": salinity; "VLP3": abundances of viral population VLP3 potentially infecting phytoplankton; []: ammonium concentrations.

## Discussion

### Autotrophic biomass along the salinity gradient

Indicators of autotrophic biomass, including chlorophyll *a *concentrations and picophytoplankton cell numbers exhibited maxima at salinities ranging between 11.0-13.0% and 8.0% and 11.0% respectively (Fig. 4). This distribution of autotrophic biomass along the lagoon is consistent with previous observations conducted in solar salterns over comparable salinity ranges [21,22,41,42]. The maximal chlorophyll *a *concentration in the present work (i.e. 14.1 μg l^-1^) was, however, well above values observed in these semi-artificial systems, which typically never exceeded 8 μg l^-1 ^[21,41,42]. In addition, maximal picophytoplankton abundance observed in the lagoon was 1.4 × 10^6 ^cells ml^-1^. This is nearly one order of magnitude higher than, the maximum abundances estimated by flow cytometry which did not exceed 3.5 × 10^5 ^cells ml^-1 ^at comparable salinities in the solar salterns of Bras del Port (Spain) [21]. These observations highlight the unique properties of the autotrophic communities found along a strong continuous salinity gradient, when compared to previous studies carried out in solar salterns.

An increase in primary producers' biomass was observed from salinity higher than 7.0% (Fig. 4) where dissolved oxygen concentrations were relatively high (Fig. 2A). This may be indicative of an enhancement of primary production in this part of the lagoon as previously observed under comparable salinity range in solar salterns [42]. An increase in primary production may be the result of a change in nutrient availability along the salinity gradient. This is consistent with the results of the BVSTEP analysis which highlighted a strong relationship between picophytoplankton abundances and ammonium concentrations (Table 2). The high abundances observed from salinity higher than 8.0% could be explained by the high ammonium concentrations found in the same part of the lagoon (Table 1). This is congruent with previous work showing the ability of picophytoplankton to efficiently utilize regenerated forms of nitrogen such as ammonium and urea [4,43]. A modification of the light regime along the salinity gradient may also have impacted the pattern of autotrophic organisms observed in the present study. The increase in suspended matter along the lagoon (Fig. 2B) is indeed likely to impact turbidity leading to a decline in light penetration in the water column in the high salinity area. However, the shallowness of the lagoon, as well as wind mixing and heating convection might prevent light limitation by ensuring sufficient turnover of the water column, as suggested by the absence of vertical stratification observed during our sampling.

Microzooplankton grazing, could also contribute to the observed pattern of autotrophic biomass along the salinity gradient. More specifically, considering the importance of microzooplankton grazing as a source of nutrients recycling in planktonic systems [44] and the high level of ammonium concentrations observed for salinity greater than 11.0% (Table 1), the sharp decrease in picophytoplankton abundances observed in the same area (Fig. 4A) may then be the result of an increase in grazing pressure. This observation is congruent with previous works reporting high microzooplankton grazing rates for salinity higher than 4.0% [42] as well as high abundances of heterotrophic nano-flagellates up to the highest salinities (i.e. > 30.0%) [45,46]. The decrease in chlorophyll *a *concentrations, representing the size fraction >1.2 μm, observed for salinity higher than 13.0% may also be the result of an increase in grazing pressure by large metazoans consumers, such as the brine shrimps (i.e. *Artemia *sp.) that were very abundant during the sampling experiment (i.e. 20-50 ind l^-1^; Seuront, unpublished data) and known to survive up to the highest salinities [41,47]. Moreover, picophytoplankton population growth is tightly controlled by fast growing protozoans consumers under high nutrients conditions [48,49] whereas larger cells are temporally and/or locally, protected from predation by slow-growing Metazoa under the same conditions [50,51]. Therefore the decrease in picophytoplankton abundances and phytoplankton biomass (size fraction>1.2 μm) observed in different part of the lagoon could be explained by a difference in size-related grazing rate along the salinity gradient (Fig. 4). However, further work is needed to fully assess the role of grazing on the distribution of autotrophic organisms along the salinity gradient.

### Picophytoplankton abundances and viruses

The peak in picophytoplankton abundance, observed for salinity ranging between 8.0% and 11.0%, was concomitant to the occurrence of the viral population VLP3 in the water column (from 5.0 to 10.0%). This viral population, exhibiting the typical cytometric signature of phytoplankton virus, was only observed in this narrow range of salinity. Considering the strong relationship between virus and their potential hosts, this result suggests a positive correlation between picophytoplankton populations and VLP3. This hypothesis is supported by the result of the BVSTEP analysis (Table 2). In addition, the abundances of VLP3 recorded in the lagoon (1.9 × 10^6 ^- 4.3 × 10^6 ^VLP ml^-1^), were in the highest range of concentrations previously reported in marine waters, i.e. >10^5 ^VLP ml^-1 ^[13]. This observation suggests an important contribution of viral lysis to picophytoplankton losses in this part of the lagoon. However, as chlorophyll concentrations were also relatively high over this salinity range (8 to 11%), VLP3 could also be infecting the larger fraction (i.e. > 1.2 μm) of the phytoplankton community. While this was beyond the objectives of the present investigation, further work will be needed to specifically assess which fraction of the phytoplankton community was infected by VLP3. Viruses infecting both components of the picophytoplankton community (i.e. cyanobacteria and eukaryotes) have been previously reported [13,52-54] and the role of viral lysis on picophytoplankton mortality is now well established [e.g. [13,14]]. However, the importance of phytoplankton viruses under this high salinity conditions has barely been investigated and further work is needed to confirm the identity of this viral population and evaluate the role of phytoplankton viruses along the salinity gradient.

### Salinity and picophytoplankton cytometric richness

The picophytoplankton cytometric-richness decreased along the salinity gradient, affecting both prokaryotic and eukaryotic picophytoplankton (Fig. 7). The existence of a decreasing trend in the number of phytoplankton species with increasing salinity has previously been observed in solar salterns ponds [21-23,41,42] and hypersaline lakes [24,55]. More specifically, a decrease in picophytoplankton cytometric-richness with increasing salinity has been reported in Bras del Port salterns [21].

The most diversified community was observed for salinity lower than 3.5% (Fig. 6). In this habitat, *Prochlorococcus *and *Synechococcus*-like populations were abundant and 5 distinct populations of pico-eukaryotes were identified. This high cytometric richness coincided with relatively low total abundance (Fig. 4B). Favourable environmental conditions may have led to the establishment of highly diversified picophytoplankton community in this brackish-marine part of lagoon. In contrast, the number of cytometrically-defined populations was limited under higher salinity conditions (Fig. 6) where the highest abundances were observed (Fig. 4B). More specifically, the peak in picophytoplankton abundances observed for salinity ranging between 8.0% and 11.0% was largely dominated by the pico-eukaryotes *P-Eu*_1 _(Fig. 6). This is consistent with previous works reporting the dominance of one population of pico- eukaryote under the same salinity range in solar salterns [21-23] and hypersaline lakes [17,24]. The existence of a bloom of pico-eukaryotes, observed in such different saline systems suggests that salinity may be the main factor triggering the dominance of pico-eukaryotes over this particular salinity range. This hypothesis is supported by the results of the BIOENV/BVSTEP analysis (Table 2). The pico-eukaryote *P-Eu*_1 _may be dominant through a higher tolerance to high salinity and the subsequent decrease in competition within the reduced picoplankton community, may have allowed this salinity-tolerant population *P- Eu*_1 _to grow extensively and flourish in this part of the lagoon. The collapse of the pico- eukaryote bloom observed for salinity greater than 11.0% was followed by an increase in *Prochlorococcus *sp. concentration which contributed to more than 40% of the total abundance for salinity greater than 15.0% (Fig. 5 and 6). This observation is consistent with many studies highlighting the abundance of cyanobacteria under extreme saline conditions [e.g. [16]]. It is also stressed that this is the first report of a *Prochlorococcus*-like population in such highly saline habitat. However, further work is therefore needed to confirm the identity of this population

The succession of picophytoplankton was only defined in the present work through their flow cytometric signature. Even if flow cytometry is a powerful tool to investigate the composition of picophytoplankton organisms, further work is needed to identify the species succeeding along the salinity gradient, especially considering the diversity existing among cyanobacteria and pico-eukaryotes [2]. However, our results provide new insight into the effect of salinity on picophytoplankton communities.

### Effect of salinity on picophytoplankton community's succession

Salinity has been identified as the main factor triggering the succession of pico-autotrophs along the salinity gradient (BIOENV/BVSTEP analyses, Table 2). Salinity could act directly on picophytoplankton assemblages by selecting groups adapted to life at a particular salt concentration. Cyanobacteria are known to tolerate and acclimate to high salt concentrations [56]. However the different groups of cyanobacteria do not exhibit the same tolerance to salinity stress and have been consequently classified into 3 groups, i.e. stenohaline, halotolerant and extremely halotolerant [57]. The decrease in cyanobacteria abundances for salinity higher than 3.5%, suggests that the populations inhabiting the brackish-marine part of the Coorong may belong to the stenohaline group with a salinity tolerance range characteristic of estuarine and marine populations. *Synechococcus *species are known to be abundant in transitional and freshwater areas [58] whereas *Prochlorococcus *species are thought to be restricted to marine waters [59]. However, the observations of *Prochlorococcus*-like populations in the Rhône River [60], in the Changjiang river estuaries [18] and in the present work in the low-salinity part of the Coorong tend to challenge this hypothesis.

In contrast, the occurrence and predominance of the pico-eukaryote *P-Eu*_1 _throughout the salinity gradient, suggest that this population may represent halotolerant organisms. The dominance of halotolerant pico-eukaryotes has been previously described for the same salinity range [17,23,24]. In particular, eukaryotic pico-autotrophs have been shown to be responsible for dense blooms in surface water of Mono Lake in California where salinity was around 8.5% [24]. The organism isolated from this lake and identified as a *Picocystis *spp., has been shown to exhibit high growth efficiency over the 0.2-15.0% salinity range, which is consistent with the range of salinity where the population *P-Eu*_1 _was found in the present work. However, this population was identified here through flow cytometry and further work is needed to unambiguously conclude on the identity of this population.

Independent of their intrinsic salinity tolerance, the succession of picophytoplankton organisms along the salinity gradient could be indirectly controlled by salinity. In particular, by controlling the diversity and abundance of microzooplankton and VLP, salinity could exert a control on the top-down processes including grazing and viral lysis. The presence of a large population of phytoplankton virus up to the highest salinity observed in the present work highlights the necessity to investigate the role of viral infection in regulating the community structure of picophytoplankton along this salinity gradient.

## Conclusions

Salinity was identified as the main factor controlling the picophytoplankton dynamic along the salinity gradient. However, the variability in nutrients availability as well as the intensity of viral lysis and microzooplankton grazing may have also played an important role in structuring the succession of picophytoplankton communities along the lagoon. The number of cytometrically-defined populations decreased with increasing salinity, affecting both prokaryotic and eukaryotic organisms. Our results also highlight the dominance of one cytometrically-defined population of pico-eukaryote throughout the salinity gradient, which was able to form a large bloom under relatively high salinity conditions (i.e. 8.0-11.0%). This finding stresses the need to further explore the specie and metabolic diversity of these small eukaryotic autotrophs along the salinity gradient.

In the literature, picophytoplankton community dynamics have been mainly described along discontinuous (i.e. solar salterns, hypersaline lakes) or weak salinity gradient. The present study constitutes the first observation of the picophytoplankton dynamics in a system where salinity continuously increased from brackish to hypersaline. Although the spatial dynamic observed here is in accordance with the patterns observed previously, the high abundance of picophytoplankton as well as the existence of a *Prochlorococcus*-like population in hypersaline waters set this saline lagoon apart from the systems studied previously. However, even if the *Synechococcus*-like and *Prochlorococcus*-like populations identified in the present work exhibited the archetypical flow cytometric signature of *Synechococcus *sp. and *Prochlorococcus *sp., further work is needed (e.g. genetic fingerprinting) to unambiguously identify these populations. The results obtained in this study provide new insight into the potential effects of salinity gradient and perturbations on phytoplankton community shifts.

## Competing interests

The authors declare that they have no competing interests.

## Authors' contributions

MS conducted the sampling experiment and data analysis and wrote the manuscript. MJB conducted the sampling experiment and ran flow cytometry analyses. TP conducted the sampling experiment. SCL conducted the sampling experiment and data analysis. LS designed the research, conducted the sampling experiment and wrote the manuscript. All authors read and approved the final manuscript.
